# Case Report: Multi-Omics Analysis and CAR-T Treatment of a Chronic Myeloid Leukemia Blast Crisis Case 5 Years After the Discontinuation of TKI

**DOI:** 10.3389/fonc.2021.739871

**Published:** 2021-09-21

**Authors:** Ya-Ru Miao, Wen Liu, Zhaodong Zhong, Yong You, Yutong Tang, Weiming Li, Xiaojian Zhu, An-Yuan Guo

**Affiliations:** ^1^Center for Artificial Intelligence Biology, Hubei Bioinformatics & Molecular Imaging Key Laboratory, Key Laboratory of Molecular Biophysics of the Ministry of Education, College of Life Science and Technology, Huazhong University of Science and Technology, Wuhan, China; ^2^Institute of Hematology, Union Hospital, Tongji Medical College, Huazhong University of Science and Technology, Wuhan, China; ^3^Department of Hematology, Tongji Hospital, Tongji Medical College, Huazhong University of Science and Technology, Wuhan, China

**Keywords:** discontinuation, blast crisis, CAR-T, TGF-β, single-cell sequencing

## Abstract

Most relapsed chronic myeloid leukemia (CML) patients after tyrosine kinase inhibitor (TKI) discontinuation are in a chronic phase and could achieve remission through restarting the TKI treatment. Here we reported a case of sudden lymphoid blast crisis after 67 months of TKI discontinuation and depicted the patient by DNA and RNA sequencing to investigate intrinsic molecular features. The mutations of TGFBR2 and PCNT and the dysregulations of TGF-β and other pathways might accelerate the B cell transformation, which may serve as a blast crisis risk indicator of CML. Single-cell transcriptome data revealed that several clusters of immature B cells and late pro-B cells presented clone evolution during the treatment. After failing multiple lines of TKIs, conditioning chemotherapies and chimeric antigen receptor T cells (CAR-T) targeting CD19 and CD22 were performed to achieve remission. In conclusion, we report the first case of a CML patient with sudden lymphoid blast crisis after a long treatment-free remission and additional gene abnormalities other than BCR-ABL1 might participate in the progression, which need to be closely monitored, and CAR-T could be a solution to the chemoresistant progression.

## Introduction

Tyrosine kinase inhibitors (TKIs) targeting *BCR–ABL1* have significantly changed the overall survival rate of chronic myeloid leukemia (CML) patients. Approximately 40% of selected patients are free from TKI and remain in this treatment-free remission (TFR) phase (1). TFR is an attractive goal for CML patients and physicians and is reported to be relatively safe. Here we report the first case of a CML patient who experienced sudden lymphoid blast crisis after 67 months of TFR. The patient was subjected to whole-genome DNA, bulk RNA, and single-cell RNA sequencing to investigate intrinsic molecular features underlying this sudden transformation. Chimeric antigen receptor T cells (CAR-T) targeting CD19 and CD22 may help the patient achieve molecular remission.

## Case Presentation

**Figure 1:**
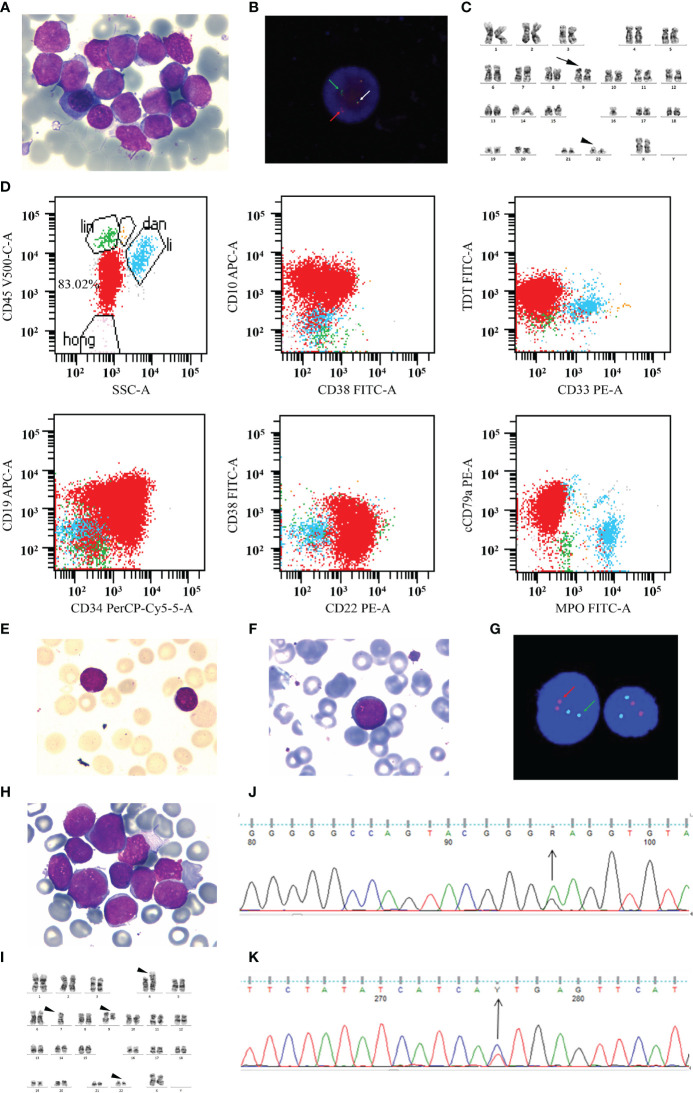
The progression of a blast crisis. **(A)** A bone marrow (BM) aspiration revealed that lymphoid blasts account for 99.5% of all nucleated cells (Wright–Giemsa, ×1,000). **(B)** Interphase FISH analysis revealed the classical 1G1R2F translocation pattern in 39% of cells before treatment. The white arrows denote fusion signals representing the BCR-ABL fusion on chromosome 22. The red arrows mark chromosome 9, and the green arrows indicate chromosome 22. **(C)** Conventional cytogenetic GTG-banding analysis indicated normal karyotype. The arrow marks chromosome 9, and the arrowhead indicates chromosome 22. **(D)** Panel of scatter plots depicting the multicolor immunophenotyping of bone marrow aspirate samples by flow cytometry. **(E)** A BM aspirate revealed that blast cells account for 9% of all nucleated cells (Wright–Giemsa, ×1,000). **(F)** A BM aspirate revealed that prolymphocytes account for 1.5% of all nucleated cells (Wright–Giemsa, ×1,000). **(G)** FISH analysis revealed that there was no abnormal signal. **(H)** BM infiltration with 94.5% of lymphoid blasts (Wright–Giemsa, ×1,000). **(I)** The conventional cytogenetic GTG-banding analysis involves multiple abnormal chromosomes. **(J, K)** The *BCR-ABL1* kinase domain mutation analysis revealed the mutation of E255K **(J)** and T315I **(K)**.

**Figure 2:**
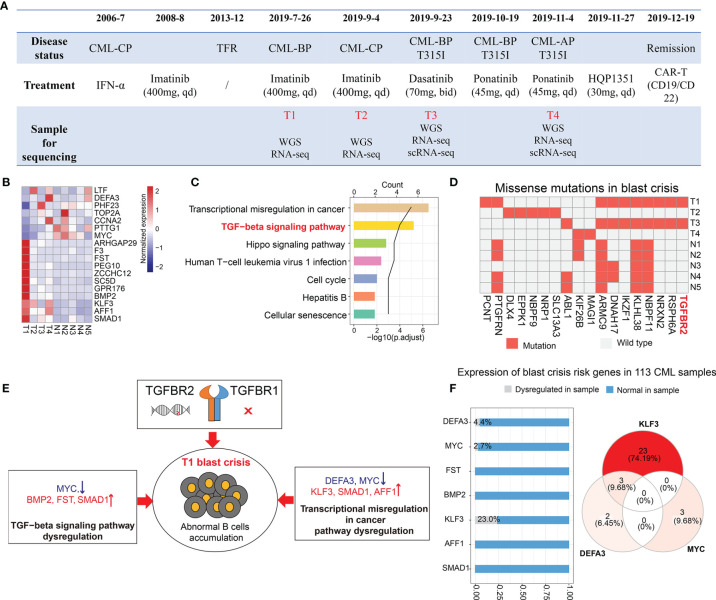
Transcriptomic and genomic analysis of the blast crisis sample. **(A)** The timeline of disease progression, treatment, and sample collection. **(B, C)** Shared differential genes in the comparisons of T1 *vs*. T3 and T1 *vs*. NCBI samples **(B)** and their enriched pathways **(C)**. **(D)** Missense mutations in our samples (T1–T4) and published blast crisis samples (N1–N5). **(E)** The possible mechanisms leading to the sudden blast crisis at T1. **(F)** The bar plot shows the proportion of chronic myeloid leukemia (CML) samples with or without the dysregulation of CML blast crisis risk genes. The Venn diagram shows the distribution of three dysregulated blast crisis risk genes (KLF3, DEFA3, and MYC) in the samples.

The patient was a 41-year-old woman diagnosed with chronic-phase CML in July 2006 and had undergone interferon-α therapy for 2 years, owing to financial difficulties. Imatinib was initiated at a standard dose in August 2008, and undetectable minimal residual disease (uMRD) was achieved after 1 month. In December 2013, 7 years after diagnosis and 5 years after MR^IS4.0^, after giving her informed consent, imatinib was stopped for clinical observation to proceed; the patient was regularly monitored (once every 3 months) after the discontinuation. On April 17, 2019, an evaluation of the *BCR–ABL1* transcript level revealed a molecular response (0.046%^IS^); a similar result (0.042%^IS^) was recorded in June 20. In July 25, the patient was admitted with symptoms of dermal ecchymosis for 3 days. The peripheral blood examination revealed a white blood cell (WBC) count of 115.2 × 10^9^/L and a platelet count of 29.0 × 10^9^/L. Imatinib (400 mg daily) was immediately reinitiated, and the WBC count gradually returned to a normal level. After about 1 week, the examination by bone marrow aspiration revealed infiltration with 99.5% of lymphoid blasts (**Figure 1A**). Interphase fluorescence *in situ* hybridization revealed the presence of a classical 1G1R2F translocation pattern in 39% of 200 cells (**Figure 1B**), and the *BCR–ABL1* transcript level was 107.10%. Conventional cytogenetic GTG-banding analysis revealed that there were no gross chromosomal abnormalities in the two metaphase cells analyzed (**Figure 1C**). Immunophenotyping indicated primitive precursor cells expressing CD19, CD34^dim^, cCD79a, CD38, CD22, CD10, and TDT at a proportion of 83.02% (**Figure 1D**). These findings confirmed the lymphoid blast crisis of CML. Then, we added the vincristine and prednisone regimen for treatment. We used this sample as the T1 sample for sequencing. On August 2, 2019, the bone marrow aspiration revealed that the proportion of blast cells was 9% (**Figure 1E**). A third bone marrow aspiration on September 4, 2019 revealed that the malignancy remained in the chronic phase (**Figure 1F**). There was no classical positive translocation pattern in 400 cells (**Figure 1G**). The *BCR–ABL1* transcript level was 5.36%. We used this sample as the T2 sample for sequencing. However, the patient entered a blast crisis again; bone marrow infiltration with 94.5% of lymphoid blasts was detected on September 23, 2019 (**Figure 1H**). The karyotype was 45,XX,der(4)t(9;22)(q34;q11)t(4:9)(p16;q22),7,der(9)del(9)(p22)t(4;9),der(22)t(9;22) (**Figure 1I**), and a mutational analysis revealed the presence of T315I and E255K mutations (**Figures 1J, K**). The *BCR–ABL1* transcript level increased to 70.43%. Based on these findings, the treatment was switched to dasatinib (140 mg daily). We used this sample as the T3 sample for sequencing. After 26 days, the treatment was switched to ponatinib (45 mg daily) by a personal way since ponatinib was not yet approved in mainland China. On November 4, 2019, the disease progressed to an accelerated phase, and the proportion of blast cells in the bone marrow was 14.5%. Only the T315I mutation was present. The *BCR–ABL1* transcript level decreased from 70.43 to 30.78%. We used this sample as the T4 sample for sequencing. However, the patient quickly became resistant to ponatinib. HQP1351 (a third-generation TKI undergoing clinical trials, 30 mg daily) administration was initiated in November 27. During December 19–21, she was conditioned with a lymphodepleting chemotherapy regimen consisting of fludarabine (25 mg/m^2^) and cyclophosphamide (20 mg/kg). Then, 4 × 10^6^ cells/kg CAR-T CD22 and 2.8 × 10^6^ cells/kg CAR-T CD19 were transfused on December 23 and 24, respectively. The *BCR–ABL1* transcript level was 0.094% on January 1, 2020. Unfortunately, this patient died of infection during the epidemic of COVID-19 in Wuhan. The entire treatment process of this patient is described in **Figure 2A**.

To investigate molecular features and find the leading cause of the sudden blast crisis at T1, we performed a comparison between the T1 sample and the other blast crisis samples, including T3 sample and public blast crisis samples from NCBI [accession IDs: PRJNA390519 (2) and PRJNA213438 (3)]. A differential expression analysis revealed 7 downregulated genes (LTF, DEFA3, PHF23, TOP2A, CCNA2, PTTG1, and MYC) and 11 upregulated genes (ARHGAP29, F3, FST, PEG10, ZCCHC12, SC5D, GPR176, BMP2, KLF3, AFF1, and SMAD1) at T1 (**Supplementary Table S1**). Most of these differential genes did not change significantly during the treatment (**Figure 2B**), which may be the risk genes of the T1 sample blast crisis. An enrichment analysis indicated that the 18 genes were mainly enriched in transcriptional misregulation in cancer, TGF-β signaling pathways, *etc.* (**Figure 2C**). The genomic analysis results showed the missense mutation of PCNT [c.4357T>G, NM_006031.6|p.(C1453G) and c.4359T>G, NM_006031.6|p.(C1453T)] only in the T1 sample (**Figure 2D**), and the BCR-ABL1 T315I mutation was shown only in the T3 sample. Interestingly, we observed a mutation on the TGF-β receptor (TGFBR2) in the T1 and T3 blast crisis samples, but not in T2, T4, and other public blast crisis samples (**Figure 2D**). TGF-β-related genes were reported to serve as regulators of B cell development and function (4). Combined with these results, we inferred that the pathway dysregulation of TGF-β and transcriptional misregulation in cancer genes and the specific mutation may play key roles in the sudden blast crisis of this patient (**Figure 2E**). After that, we observed the expression of blast crisis risk genes in 113 CML patients from another cohort. The dysregulation of KLF3, DEFA3, or MYC was observed in 31 CML patients (**Figure 2F**), and three patients, who may have a greater blast crisis risk, were observed with a dysregulation of both KLF3 and DEFA3.

**Figure 3:**
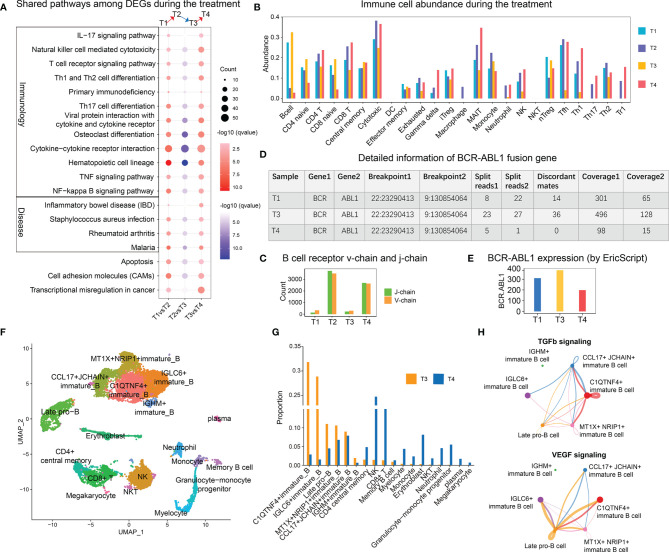
Transcriptome features relevant to treatment and disease progression. **(A)** Shared enriched pathways with differentially expressed genes during disease progression and treatment. The red color indicates the pathway enriched with upregulated genes [T1 *vs*. T2 (up) and T3 *vs*. T4 (up)], whereas the blue color indicates the pathway enriched with downregulated genes [T2 *vs*. T3 (down)]. **(B)** The distribution of immune cell abundance during disease progression and treatment. **(C)** The abundance of B cell receptor V-chain and J-chain per sample. **(D)** Detailed information on the BCR–ABL1 fusion gene in each sample. **(E)** Expression of *BCR–ABL1* in the T1, T3, and T4 samples. **(F)** Two-dimensional uniform manifold approximation and projection visualization of the cellular composition of the T3 and T4 samples. **(G)** Bar plot of the cell proportions of the T3 and T4 samples. **(H)** Circle plot showing the inferred TGF-β and VEGF signaling networks. The size of the dot represents the cell number. A line connecting two cell types indicates a receptor–ligand interaction between them. The thickness of the line represents the signal intensity.

Next, we explored the dynamic change of the molecular features during the treatment. A total of 19 pathways were shared by differential genes during treatment and disease progression (**Figure 3A**), and most of these pathways were related to immunology. The immune cell abundance analysis by ImmuCellAI (5) revealed that the T1 and T3 samples contained higher numbers of B cells and naive T cells (**Figure 3B**); however, the diversity of their B cell receptor was rare (**Figure 3C**). After treatment, the number of cells with cytotoxic and helper function was increased, whereas that of B cells was reduced (**Figure 3B**). Subsequently, a gene fusion analysis revealed the presence of the BCR–ABL1 fusion in T1, T3, and T4 samples (**Figure 3D**), of which T3 had the highest fusion expression (**Figure 3E**).

The single-cell RNA-seq analysis revealed the substantial accumulation of several clusters of immature B cells and late pro-B cells in the T3 sample (**Figures 3F, G**), while the T4 sample had the highest proportion of NK and CD8+ T cells. A cell communication analysis revealed that CCL17^+^ JCHAIN^+^ and MT1X^+^ NRIP1^+^ immature B cells, whose numbers slightly decreased after treatment, were mainly involved in TGF-β signaling (**Figure 3H**). Late pro-B cells and IGLC6+ immature B cells were mainly involved in VEGF signaling.

## Discussion

More than 95% of CML patients who experience a relapse after discontinuation could achieve a molecular response after restarting the TKI treatment, and 90% of them could return to uMRD (6), indicating that the cessation might be safe. However, three cases of lymphoid blast crisis have been reported in patients who resumed the TKI treatment after an unsuccessful attempt (7–9). In this study, we reported a unique case of a patient who suffered sudden blast crisis during the remission period, which was different from the former three cases whose transformation occurred after a relapse. As a result, the concept of safe drug withdrawal remained subject to further assessment, as long-lasting TFR did not guarantee a progression-free survival. However, the rate was significantly lower than that of patients receiving imatinib or nilotinib for more than 5 years but still exhibiting disease progression (10). Recently, we reported that the SNP rs139130389 in FLOR3 is an indicator of TFR (11), and the said SNP was not observed in this patient.

Genome and transcriptome analyses revealed that the presence of TGFBR2 mutation and pathway dysregulation, such as TGF-β, might accelerate B cell transformation and lead to the sudden blast crisis of T1, which may serve as indicators of CML blast crisis. Besides this, a research by Pagani et al. (12) revealed that MRD was identified predominantly in the lymphoid compartment and never in the granulocytes, which suggests that the detection of MRD should be accurate down to the cell lineage level. In this work, our scRNA-seq analysis showed that several immature B cell and late pro-B cell subtypes were accumulated during a relapse and decreased after treatment. On the contrary, MT1X+NRIP1+ immature B cell and CCL17+JCHAIN+ immature B cell were residual after treatment, which might be the MRD source and root for disease progression. These B cells may not only be driven by BCR-ABL1; thus, multiple TKIs were tried but failed to eliminate. However, as they carried CD19 and CD22 markers, targeted CAR-T was performed, and major molecular remission was achieved after infusion. Unfortunately, all laboratory departments were closed during the epidemic of COVID-19 in Wuhan, and we were unable to keep on monitoring after the CAR-T infusion. It has been reported that CAR-T could overcome the cytogenetic or molecular abnormalities of relapsed and refractory B cell malignancies (13, 14). Thus, we confirm the cognition that immunotherapy such as CAR-T may provide more effective options in blast crisis CML after cessation.

## Data Availability Statement

The datasets presented in this study can be found in online repositories. The names of the repository/repositories and accession number(s) can be found below: HRA001152, https://bigd.big.ac.cn/gsa-human/browse/HRA001152.

## Ethics Statement

The studies involving human participants were reviewed and approved by the Medical Ethics Committee of the Department of Hematology, Tongji Hospital, Tongji Medical College, Huazhong University of Science and Technology (TJ-IRB20160310). The patient gave her written informed consent in accordance with the Declaration of Helsinki. This study was registered at www.chictr.org.cn as ChiCTR-OPN-16008526. The patients/participants provided their written informed consent to participate in this study. Written informed consent was obtained from the individual(s) for the publication of any potentially identifiable images or data included in this article.

## Author Contributions

Y-RM and WLiu performed formal analysis and wrote the original draft. XZ and A-YG conceptualized and designed the research, wrote, reviewed, and edited the manuscript, and funded and supervised the study. ZZ, YY, WLi, and YT provided assistance on sample and experiments. All authors contributed to the article and approved the submitted version.

## Funding

This work was supported by the National Natural Science Foundation of China (grant numbers 31822030, 31771458, and 81873440), the Excellent Young Scientist Foundation of Tongji Hospital (grant number 2020YQ0012), and the Key R&D Plan of Hubei Province (grant numbers 2020BCB021 and 2020BCB043).

## Conflict of Interest

The authors declare that the research was conducted in the absence of any commercial or financial relationships that could be construed as a potential conflict of interest.

## Publisher’s Note

All claims expressed in this article are solely those of the authors and do not necessarily represent those of their affiliated organizations, or those of the publisher, the editors and the reviewers. Any product that may be evaluated in this article, or claim that may be made by its manufacturer, is not guaranteed or endorsed by the publisher.
