# Supplementary material for: Case Report: Multi-Omics Analysis and CAR-T Treatment of a Chronic Myeloid Leukemia Blast Crisis Case 5 Years After the Discontinuation of TKI
Source: Front Oncol. 2021 Sep 21;11:739871. doi: 10.3389/fonc.2021.739871 (PMC8490701; doi:10.3389/fonc.2021.739871)
Supplement: Supplementary file 1 [file DataSheet_1.docx]

Supplementary Materials for

Multi-omics data analysis of a chronic myeloid leukemia blast crisis case 5 years after the discontinuation of TKI

Ya-Ru Miao^1†^, Wen Liu^2†^, Zhaodong Zhong^2^, Yong You^2^, Yutong Tang^2^, Weiming Li^2^, Xiaojian Zhu^3*^, An-Yuan Guo^1*^

Correspondence to: ([zhuxiaojian@hust.edu.cn](mailto:zhuxiaojian@hust.edu.cn)) and guoay@hust.edu.cn

^1^Center for Artificial Intelligence Biology, Hubei Bioinformatics & Molecular Imaging Key Laboratory, Key Laboratory of Molecular Biophysics of the Ministry of Education, College of Life Science and Technology, Huazhong University of Science and Technology, Wuhan, China

^2^Institute of Hematology, Union Hospital, Tongji Medical College, Huazhong University of Science and Technology, Wuhan, China

^3^Department of Hematology, Tongji Hospital, Tongji Medical College, Huazhong University of Science and Technology, Wuhan, China

These authors contributed equally to this work: Ya-Ru Miao & Wen Liu.

This PDF file includes:

Materials and Methods

Table S1

**Materials and Methods**

1. Sample collection

We collected the patient's clinical follow-up data and laboratory results after blast crisis, including bone marrow cytology, interphase FISH analysis, conventional cytogenetic GTG-banding analysis, multicolor immunophenotyping and BCR-ABL1 kinase domain mutation analysis. Using standard operating procedures, mononuclear cells from bone marrow aspirates were isolated using Ficoll density gradient separation and cryopreserved in 90% FBS/10%DMSO for storage in liquid nitrogen. Furthermore, PBMC samples of 113 CML patients were collected from Institute of Hematology of Union Hospital.

1. Next generation sequencing

The RNA quality was determined with the Agilent 4200 Bioanalyzer. RNA-seq library was prepared according to Illumina’s TruSeq protocol. The library was sequenced on the Novaseq 6000 platform with the 2 × 150 bp paired-end strategy at HGC (Shenzhen, China). For WGS, we used the Agilent V6 kit for 150-bp paired-end sequencing on Hiseq x ten platform at HGC. Single-cell libraries were prepared from freshly isolated PBMCs by using 10Xgenomics DNA V3 Reagent Kits. The cells and kit reagents were mixed with gel beads containing barcoded oligonucleotides (UMIs) and oligo dTs (used for reverse transcription of polyadenylated RNAs) to form reaction vesicles called gel bead-in-emulsions (GEMs). The barcoded cDNAs in each GEM were pooled for PCR amplification, and adapter and sample indices were added. Single-cell libraries were sequenced with 150bp paired-end reads on the Illumina Nova6000b platform, with mostly 1 sample per lane.

1. Transcriptome data analysis

Transcript reassembly and quantification were processed according to the HISAT2-StringTie-ballgown pipeline^1^ with Ensembl v83 (for protein-coding genes) and NONCODE v6 (for lncRNAs) annotations^2^. The resulting transcripts were pooled across samples using the merge function of StringTie^3^, discarding all the redundant isoforms. The abundance of genes was estimated by StringTie and enumerated using FPKM (fragments per kilobase of transcript per million mapped reads). Genes with FPKM > 1 were kept for further analysis. Differentially expressed gene (DEG) analysis was performed using NOISeq^4^ with the significance threshold (probability > 0.9 and |fold change| > 2). Besides, raw data of other reported blast crisis samples were collected from the published data by accession id PRJNA390519^5^ and analyzed using the same pipeline described above.

Gene enrichment analysis was performed by R package clusterProfile^6^. Gene-based pathway crosstalk analysis was performed using ClueGO^7^. The TF gene list was downloaded from AnimalTFDB 3.0^8^. PPI network was analyzed on STRING v11^9^ web tool. Immune cell abundance per sample were calculated by ImmuCellAI^10^ web tool. Gene fusion was predicted by Arriba (<https://github.com/suhrig/arriba>). BCR was detected by self-build tool CATT^11^. Other visualizations were implemented using R language and its packages, such as gglot2 and heatmap.

1. Whole-genome data analysis

Raw sequence reads from WGS sequencing were transformed to bam format file by using tools packed in GATK4^12^ (Genome Analysis Toolkit, v4) in the following steps: (1) Raw sequence reads were mapped to reference human genome GRCh38. (2) Mark duplicated step was performed to mitigate biases introduced by data generation steps. (3) Recalibrate the base quality scores to improve the accuracy of variant calling step. Next, MuTect2^13^ was used to generate mutations using default parameters by comparing BAM files from tumor sample with background mutations. Finally, all mutations were annotated by using Ensembl variant effect predictor (VEP)^14^. CNV detection in samples was performed using CNVkit^15^.

1. Single cell data analysis

The analysis pipelines in Cell Ranger (https://github.com/10XGenomics/cellranger) were used for single cell sequencing data processing. FASTQ files were generated using cellranger mkfastq with default parameters. Then, cellranger count was mapped on the human genome (CRCH38/hg38) using STAR^16^ and UMIs were counted for each gene. The outputs of cellranger count for individual samples were integrated using cellranger “aggr” function, in which read depths are normalized based on the confidently mapped reads. Then Seurat^17^ was used to further analyzing the data produced by CellRanger. The first step was quality control, which filter cells with gene expression, unique UMI and percent of mitochondrial gene. Then, principal component analysis (PCA), tSNE, and UMAP clustering algorithms were used to visualize clustered cells in 2D space. Interaction among immature B cells was analyzed by CellChat^18^, which is a tool can quantitively infer and analyze intercellular communication networks from scRNA-seq data. CellChat predicts major signaling (receptor-ligand) for cells and how those cells and signals coordinate for functions using networks. Immature B cells from T3 and T4 samples were taken as input of CellChat. Then, cell communications among cells were inferred by evaluating expression of receptor and ligand of these cells. And interactions correlated with CML were presented in the result.

1. Blast crisis samples from NCBI

RNA-seq sequencing and whole genome sequencing blast crisis samples from published data were collected by accession ID PRJNA390519^5^ and PRJNA213438^19^ in NCBI, respectively.

**References**

1. Pertea, M., Kim, D., Pertea, G. M., Leek, J. T. & Salzberg, S. L. Transcript-level expression analysis of RNA-seq experiments with HISAT, StringTie and Ballgown. *Nature Protocols* **11**, 1650–1667 (2016).

2. Zhao, Y. *et al.* NONCODE 2016: an informative and valuable data source of long non-coding RNAs. *Nucleic Acids Res.* **44**, D203-208 (2016).

3. Pertea, M. *et al.* StringTie enables improved reconstruction of a transcriptome from RNA-seq reads. *Nat Biotechnol* **33**, 290–295 (2015).

4. Tarazona, S., García, F., Ferrer, A., Dopazo, J. & Conesa, A. NOIseq: a RNA-seq differential expression method robust for sequencing depth biases. *EMBnet.journal* **17**, 18–19 (2011).

5. Li, S.-Q. *et al.* Transcriptome profiling reveals the high incidence of hnRNPA1 exon 8 inclusion in chronic myeloid leukemia. *J Adv Res* **24**, 301–310 (2020).

6. Yu, G., Wang, L.-G., Han, Y. & He, Q.-Y. clusterProfiler: an R package for comparing biological themes among gene clusters. *OMICS* **16**, 284–287 (2012).

7. Bindea, G. *et al.* ClueGO: a Cytoscape plug-in to decipher functionally grouped gene ontology and pathway annotation networks. *Bioinformatics* **25**, 1091–1093 (2009).

8. Hu, H. *et al.* AnimalTFDB 3.0: a comprehensive resource for annotation and prediction of animal transcription factors. *Nucleic Acids Res* **47**, D33–D38 (2019).

9. Szklarczyk, D. *et al.* STRING v11: protein–protein association networks with increased coverage, supporting functional discovery in genome-wide experimental datasets. *Nucleic Acids Res* **47**, D607–D613 (2019).

10. Miao, Y.-R. *et al.* ImmuCellAI: A Unique Method for Comprehensive T-Cell Subsets Abundance Prediction and its Application in Cancer Immunotherapy. *Advanced Science* **7**, 1902880 (2020).

11. Chen, S.-Y., Liu, C.-J., Zhang, Q. & Guo, A.-Y. An ultra-sensitive T-cell receptor detection method for TCR-Seq and RNA-Seq data. *Bioinformatics* (2020) doi:10.1093/bioinformatics/btaa432.

12. McKenna, A. *et al.* The Genome Analysis Toolkit: a MapReduce framework for analyzing next-generation DNA sequencing data. *Genome Res.* **20**, 1297–1303 (2010).

13. Cibulskis, K. *et al.* Sensitive detection of somatic point mutations in impure and heterogeneous cancer samples. *Nature Biotechnology* **31**, 213–219 (2013).

14. McLaren, W. *et al.* The Ensembl Variant Effect Predictor. *Genome Biology* **17**, 122 (2016).

15. Talevich, E., Shain, A. H., Botton, T. & Bastian, B. C. CNVkit: Genome-Wide Copy Number Detection and Visualization from Targeted DNA Sequencing. *PLOS Computational Biology* **12**, e1004873 (2016).

16. Dobin, A. *et al.* STAR: ultrafast universal RNA-seq aligner. *Bioinformatics* **29**, 15–21 (2013).

17. Stuart, T. *et al.* Comprehensive Integration of Single-Cell Data. *Cell* **177**, 1888-1902.e21 (2019).

18. Jin, S. *et al.* Inference and analysis of cell-cell communication using CellChat. *bioRxiv* 2020.07.21.214387 (2020) doi:10.1101/2020.07.21.214387.

19. Sabri, S., Keyhani, M. & Akbari, M. T. Whole Exome Sequencing of Chronic Myeloid Leukemia Patients. *Iran. J. Public Health* **45**, 346–352 (2016).

**Table S1 Expression (TPM) of shared up- and down- regulated genes in the comparison of T1 with other CML samples**

|  | T1 | | T3 | N1 | N2 | N3 | N4 | N5 |
| --- | --- | --- | --- | --- | --- | --- | --- | --- |
| LTF | 16.69 | 161.57 | | 86.37 | 374.54 | 95.97 | 521.51 | 1883.74 |
| DEFA3 | 10.22 | 236.12 | | 1413.95 | 377.84 | 767.86 | 2028.08 | 8360.88 |
| PHF23 | 4.59 | 44.38 | | 18.98 | 25.76 | 27.98 | 22.82 | 21.58 |
| TOP2A | 9.17 | 112.16 | | 26.22 | 318.66 | 42.94 | 33.74 | 65.53 |
| PTTG1 | 12.23 | 49.78 | | 198.28 | 160.99 | 88.19 | 48.62 | 149.10 |
| MYC | 9.10 | 34.17 | | 61.77 | 77.74 | 57.01 | 45.96 | 20.80 |
| ARHGAP29 | 210.33 | 23.19 | | 0.13 | 0.12 | 0.06 | 0.20 | 0.02 |
| F3 | 36.49 | 1.07 | | 0.35 | 0.34 | 3.25 | 0.05 | 0.36 |
| FST | 25.89 | 0.48 | | 0.32 | 1.28 | 0.33 | 0.00 | 0.84 |
| PEG10 | 151.44 | 7.39 | | 0.05 | 0.24 | 0.22 | 0.01 | 0.08 |
| ZCCHC12 | 46.20 | 0.32 | | 0.00 | 0.00 | 1.49 | 0.00 | 0.07 |
| SC5D | 147.00 | 12.37 | | 4.37 | 11.08 | 14.22 | 7.20 | 3.73 |
| GPR176 | 192.98 | 18.69 | | 0.01 | 0.32 | 0.09 | 0.10 | 0.20 |
| BMP2 | 106.65 | 9.35 | | 0.00 | 1.47 | 0.12 | 0.20 | 2.02 |
| KLF3 | 159.88 | 15.14 | | 14.92 | 24.76 | 34.76 | 7.98 | 11.04 |
| AFF1 | 145.83 | 61.38 | | 14.41 | 25.61 | 30.35 | 7.97 | 9.29 |
| SMAD1 | 99.32 | 35.74 | | 1.38 | 0.79 | 26.99 | 7.33 | 2.25 |
